# Host-specific and environmental core bacteria differentially shape the stability and function of the *Sphagnum* phyllosphere

**DOI:** 10.1093/ismeco/ycaf221

**Published:** 2025-11-25

**Authors:** Xiangbo Yin, Lee Ping Ang, Rui-Liang Zhu, Hamed Azarbad, Hai-Hang Ni, Meng-Lu Chai, Changqing Liu, Fanhao Kong, Ling-Juan Liu, Sheng-Long Liu, Yi Ma, Han-Di Zhou, Zhen-Sha Luo, Xue-Kai He, Li-Xin Ye, Hao Li, Lei Shu

**Affiliations:** Bryology Laboratory, School of Life Sciences, East China Normal University, Shanghai 200241, China; Department of Geography, Ecological Plant Geography, University of Marburg, Marburg 35032, Germany; Guangdong Provincial Key Laboratory of Biotechnology for Plant Development, School of Life Sciences, South China Normal University, Guangzhou 510631, China; Bryology Laboratory, School of Life Sciences, East China Normal University, Shanghai 200241, China; Institute of Advanced Agricultural Science and Technology, East China Normal University, Shanghai 200062, China; Shanghai Institute of Eco-Chongming (SIEC), East China Normal University, Shanghai 200062, China; Department of Biology, Evolutionary Ecology of Plants, Philipps University Marburg, Marburg 35043, Germany; Bryology Laboratory, School of Life Sciences, East China Normal University, Shanghai 200241, China; Bryology Laboratory, School of Life Sciences, East China Normal University, Shanghai 200241, China; Department of Biochemistry & Synthetic Metabolism, Max Planck Institute for Terrestrial Microbiology, Karl-von-Frisch Straße 10, 35043 Marburg, Germany; Department of Geography, Ecological Plant Geography, University of Marburg, Marburg 35032, Germany; Longquan Nature Conservation Center of Baishanzu National Park, Zhongshan West Road No. 80, Longquan, Lishui, Zhejiang Province 323700, China; Longquan Nature Conservation Center of Baishanzu National Park, Zhongshan West Road No. 80, Longquan, Lishui, Zhejiang Province 323700, China; Longquan Nature Conservation Center of Baishanzu National Park, Zhongshan West Road No. 80, Longquan, Lishui, Zhejiang Province 323700, China; Longquan Nature Conservation Center of Baishanzu National Park, Zhongshan West Road No. 80, Longquan, Lishui, Zhejiang Province 323700, China; Longquan Nature Conservation Center of Baishanzu National Park, Zhongshan West Road No. 80, Longquan, Lishui, Zhejiang Province 323700, China; Longquan Nature Conservation Center of Baishanzu National Park, Zhongshan West Road No. 80, Longquan, Lishui, Zhejiang Province 323700, China; Longquan Nature Conservation Center of Baishanzu National Park, Zhongshan West Road No. 80, Longquan, Lishui, Zhejiang Province 323700, China; Bryology Laboratory, School of Life Sciences, East China Normal University, Shanghai 200241, China; Institute of Advanced Agricultural Science and Technology, East China Normal University, Shanghai 200062, China; Shanghai Institute of Eco-Chongming (SIEC), East China Normal University, Shanghai 200062, China; Bryology Laboratory, School of Life Sciences, East China Normal University, Shanghai 200241, China; Institute of Advanced Agricultural Science and Technology, East China Normal University, Shanghai 200062, China; Shanghai Institute of Eco-Chongming (SIEC), East China Normal University, Shanghai 200062, China

**Keywords:** peat moss, core bacterial microbiome, plant-microbial interaction, environmental filtering, microbial stability, subtropical mountain

## Abstract

*Sphagnum* mosses maintain peatland ecosystem stability through intimate associations with their microbiomes. As the foundational component of these communities, the core microbiome enables ecosystems to resist, absorb, and recover from environmental changes, yet the roles and processes of *Sphagnum* core members remain poorly understood, particularly in subtropical ecosystems. Here, we identified different components of core microbiomes and found that host-specific and environmental core microbiomes differentially shape the stability and function of *Sphagnum* phyllosphere bacteria by examining vertical stratification within a litter–*Sphagnum*–soil system in a subtropical mountain forest. *Sphagnum* harbors a microbial community that is significantly distinct from its surrounding environment (i.e. litter and soil), with community assembly primarily driven by deterministic processes, whereas litter and soil communities are more strongly shaped by stochastic processes. *Sphagnum* host-specific core taxa, enriched in carbon- and nitrogen-cycling lineages (i.e. *Ca.* Eremiobacterota), stabilized microbial composition, whereas environmental core taxa enhanced interaction strength and network robustness, and these groups responded differently to environmental filters (e.g. pH and elevation). Our framework highlights that core microbiomes are not functionally homogeneous, but instead reflect contrasting strategies that collectively shape ecosystem stability*.*

## Introduction

*Sphagnum* mosses are foundational biological engineers that modulate pH, retain water, and regulate nutrient cycling across the ecosystems they dominate. In boreal and subalpine peatlands, *Sphagnum* creates and maintains saturated, acidic, and oxygen-poor conditions that inhibit decomposition and promote long-term carbon sequestration [1, 2]. In forested ecosystems where *Sphagnum* carpets dominate the ground layer, it enhances phosphorus retention and mediates nutrient dynamics, influencing tree root access and belowground interactions [3, 4]. These ecosystem functions are closely linked to diverse *Sphagnum*-associated microbiomes, particularly phyllosphere communities involved in carbon cycling, nitrogen fixation, and methane oxidation [5, 6]. For example, nitrogen-fixing cyanobacteria in the *Sphagnum* phyllosphere can convert atmospheric N₂ into ammonium via nitrogenase activity and transfer up to 35% of the fixed nitrogen to *Sphagnum* in exchange for photosynthates [7]. As key drivers of biogeochemical processes, these microbial partners play a central role in the functional stability and resilience of *Sphagnum*-dominated ecosystems under global change.

However, most microbiome studies have focused primarily on *Sphagnum* tissues, often overlooking the surrounding microbial habitats such as litter and soil [5, 8]. This narrow ecological scope limits our ability to determine whether *Sphagnum* serves as a distinct microbial habitat shaped by active host filtering or whether the observed assemblages simply reflect passive dispersal from the surrounding environment. Such knowledge gap is more evident in (sub)tropical *Sphagnum*-dominated habitats, which are often located in mountainous regions and face heightened exposure to warming, shifting precipitation regimes, and direct human pressures such as deforestation, tourism and drainage [9–11]. These stressors may interact to disrupt microbial processes across the microhabitats formed by *Sphagnum*, and its immediate surroundings, including the overlying litter and underlying soil [12, 13]. Therefore, studying the litter–*Sphagnum*–soil vertical system is crucial to disentangle the ecological drivers of microbiome assembly, distinguish host-specific from environment-derived taxa, and understand how interactions between *Sphagnum* and its neighboring habitats influence ecosystem function and resilience under global change.

The core microbiomes, typically defined as the set of microbial taxa consistently shared across individuals of a host species, is often assumed to reflect functionally important relationships such as contributions to host nutrition, defense, or stress tolerance [13–15]. However, this assumption has increasingly been challenged by evidence showing that core microbiomes are rarely conserved across large spatial scales and among distinct habitats [16–19]. Host-associated microbiomes are shaped by combination of deterministic forces, such as host filtering, environmental selection, and stochastic processes like dispersal and ecological drift [20–22]. As a result, core microbiomes may contain a mix of functionally relevant, host-selected taxa and environmentally derived microbes that consistently appear through passive dispersal.

Yet, not all core members are ecologically equivalent. The central challenge is not simply to define core microbiomes taxonomically or functionally, but to distinguish true host-associated members from taxa shared through consistent environmental exposure. Overlooking this internal heterogeneity can obscure links between core microbiomes and host function, especially when non–host-selected taxa dominate the core composition and introduce ecological “noise,” i.e. variation in community composition or function driven by environmental carryover rather than genuine host selection [23–25]. Disentangling these components is essential for understanding how different core microbiomes contribute to host fitness and broader ecosystem functioning. However, this ecological stratification remains largely unexplored—especially in natural ecosystems where host–environment boundaries are complex and subtle.

Furthermore, microbial community stability, defined as the ability to maintain community structure and function in response to environmental change, is fundamental to ecosystem resilience and long-term sustainability under global change [26]. Emerging evidence highlights the pivotal role of core microbiomes in maintaining community stability [27, 28]. Core taxa often act as ecological anchors, contributing disproportionately to microbial network structure, redundancy, and stress buffering capacity, thereby enhancing resistance and recovery after perturbations [29]. Understanding the *Sphagnum* core microbiomes is thus not only essential for identifying stable and functionally important microbial groups for *Sphagnum* communities but also predicting how their dominated ecosystems (e.g. peatland and subalpine forests) and microbial ecosystems respond to environmental change.

Here, we take advantage of the vertically organized litter–*Sphagnum*–soil system in a subtropical mountain ecosystem of the Baishanzu Nature Reserve, China, as a model to address this gap. This microstratified environment presents an ideal context to dissect how vertical habitat gradients shape bacterial communities. *Sphagnum* not only forms the ecological and structural center of the system but also mediates resource and microbial flow across layers, offering a powerful system to explore microbiome assembly, divergence, and function. In this study, we aim to (i) characterize the diversity, composition, and functional potential of microbiomes across vertical habitat strata; (ii) redefine the *Sphagnum* core microbiome by separating host-specific and environmental core taxa; and (iii) test the ecological and functional contributions of different core groups to community stability and predicted metabolic function. By doing so, this study offers a new ecological insight on phyllosphere core microbiomes, with implications for host–microbe evolution, and ecosystem management in subtropical ecosystems.

## Materials and methods

### Study site description and field sample

This study was conducted at Baishanzu National Nature Reserve (27.83°N, 119.18°E), a typical subtropical monsoon zone climate nature reserve located in Qingyuan county, China (Fig. S1). The region has a subtropical climate with an average annual temperature of 12.8°C, annual precipitation of 2341.8 mm, and relative humidity of 84.0%. The region’s complex topography, humid temperate climate, and pronounced altitudinal gradient have shaped the most representative subtropical forest ecosystem in the Yangtze River Delta, noted for its high national authenticity and ecological integrity [30, 31]. Five *Sphagnum*-dominated sites along an elevational gradient were selected in the nature reserve (Fig. S1): (A) Highway-adjacent (27.9098°N,119.2037°E, *Sphagnum* carpet at forest edges); (B) Fengbai ancient trail (27.9009°N, 119.1891°E, *Sphagnum* carpet inside forest); (C) Grand Canyon Waterfall (27.8778°N, 119.1770°E, *Sphagnum* carpet on the cliff); (D) Longquan Grand Canyon’s shaded side (27.9112°N, 119.1753°E, *Sphagnum* carpet inside forest); (E) Fengyang lake (27.8731°N, 119.1853°E, *Sphagnum* carpet in the peatland). *Sphagnum junghuhnianum* Dozy & Molk and *Sphagnum palustre* L. are the main *Sphagnum* species in the five sites.

In October of 2024, five colonies (i.e. a group of shoots of one species that live and interact closely with each other) of *Sphagnum* were randomly selected in each site. Each colony was used as one plot, and the distance between plots was over 50 m. At each plot, we collected above leaf litter, top fresh parts from at least 10 shoots, below organic soil. For *Sphagnum* samples, debris and other plant materials were removed in the field, and only the top green portions (0–3 cm from the shoot tip) were collected to minimize contamination from soil particles [32]. Leaf litter was collected from five different points above each *Sphagnum* colony, and organic soil was sampled from the ground surface (top 0–2 cm). All *Sphagnum*, leaf litter, organic soil samples from one plot were pooled in a sterile plastic roll bag, respectively, and stored on ice in a cooler until reaching the laboratory, where samples were stored at −20°C until further processing. In total, 75 samples, including 25 of leaf litter, 25 of *Sphagnum*, and 25 of soil, were collected from the 25 plots and five sites. In addition, elevation and pH values were measured at each colony in the field.

### DNA extraction, library preparation, and sequencing

The DNA extraction process followed the protocol of Kembel *et al*. [33], with some modifications. To obtain epiphytic microbial cells, *Sphagnum*, litter, and soil samples were placed into sterile 50-ml Falcon tubes and phosphate buffered saline supplemented with Tween (PBST) 20 was poured into the tube to cover the shoots (30–35 ml). Samples were removed from the tube after shaking on a vortex for 30 s, then on a stirrer plate for 5 min. Microbial cells were collected from leaf surfaces and separated from the PBST saline by centrifuging at 4000×*g* for 20 min at 4°C [34]. The supernatant was discarded and the pellet was transferred to a PowerBead tube of the DNeasy Power Soil DNA Isolation Kit (Qiagen, Germany) for DNA extraction, following the manufacturer’s instructions. Bacterial communities were characterized by amplifying and sequencing the V4–V5 regions of the nuclear 16S ribosomal RNA gene with the primer pair 515F-Y (5′-GTGYCAGCMGCCGCGGTAA-3′)/926R (5′-CCGYCAATTYMTTTRAGTTT-3′) [35]. Library preparation for Illumina sequencing was performed according to the manufacturer’s instructions for user-defined primers. The first amplification was performed in a 25-μl reaction mix composed of 9 μl of UltraPure™ DNase/RNase-Free distilled water (GIBCO, Life Technologies), 200 μM of each dNTP, 1.5 mM of Mg^2+^, 200 nM of each primer, 1 U of HotStarTaq Plus DNAPolymerase (Qiagen, Valencia, CA), and 2.5 μl of DNA extract. Thermocycling conditions were as follows: initial denaturation step at 95°C for 5 min; 34 cycles at 94°C for 30 s, 50°C for 30 s, and 72°C for 1 min; and a final elongation step at 72°C for 10 min. PCR products were purified with magnetic beads (Agencourt AMPure XP). Sequencing was performed on an Illumina MiSeq platform with a PE300 Platform of the Majorbio Bio-Pharm Technology Co. Ltd. (Shanghai, China).

### Bioinformatic processing

The DADA2 Pipeline (1.16 version for 16S Pipeline Workflow) on R platform was then used to exclude primer sequences, filter and de-noise sequences, de-replicate unique amplicon sequence variants (ASVs, similar to 100%-identity operational taxonomic units), and remove chimeric sequences [36]. Those ASVs were classified (minBoot = 80) with the SILVA taxonomic databases version 138.2 for 16S [37]. Then, we identified and removed non-bacterial ASVs from bacterial ASV tables including ASVs classified as Archaea, Mitochondria, Chloroplast, Eukaryote, Archaea, and Rickettsioses. After the processing, 27 550 ASVs remained for bacteria (12 463 ASVs for *Sphagnum* samples; 16 578 ASVs for soil samples; 12 482 ASVs for litter samples).

The tables generated by the DADA2 Pipeline were imported into R as a phyloseq object (phyloseq package [38]) for further quality filtering steps. The removal of sequences identified as contaminants was used by the decontam package [39], but declined with elevation, likely due to elevation-d [39], and then singletons, doubletons, and ASV with <10 reads were removed to control for the potential influence of rare sequences. The number of sequences used from each sample was rarefied to the minimum sample size, 13 095 ASVs per sample, for further analysis-driven shifts in host functional traits [40].

### Statistical analyses

All analyses were performed with the statistical platform R 4.4.3 with R Studio software [41]. Results were visualized with the ggplot2 package version 4.0.0 [42]. All *Sphagnum* samples were analyzed together rather than by species to reflect their natural growth pattern and support the study’s objective of identifying general microbial patterns in *Sphagnum* as a unified habitat. In the subtropical habitats studied, *Sphagnum* species commonly co-occur in mixed mats, making species-level separation ecologically and practically unnecessary. A preliminary survey (not published yet) conducted in spring 2024 confirmed that microbial community composition did not differ significantly among co-occurring *Sphagnum* species, but did differ markedly from adjacent litter and soil. This supports a habitat-based approach and provides a robust basis for combining *Sphagnum* samples in all statistical analyses. Analyses were designed as follows to address three core research objectives:

#### Characterizing microbial diversity, composition, and functional potential across the vertical litter–*Sphagnum*–soil system in subtropical peatlands

To quantify differences in microbial community properties across habitats (i.e. leaf litter, *Sphagnum*, and soil), we assessed a suite of diversity, phylogenetic, network, and functional metrics, including observed richness, inverse Simpson index, phylogenetic diversity (PD), net relatedness index (NRI), positive and negative cohesion, carbon and energy metabolism, and stress adaptation and ecosystem resilience. PD and NRI were calculated from a pruned bacterial phylogeny using the picante package version 1.8.2 [43, 44]. Co-occurrence networks were constructed using Spearman’s rank correlations among ASV relative abundances after compositional data normalization. Positive and negative cohesion values—representing the summed strengths of positive (cooperative) and negative (competitive) interactions—were then derived from the resulting networks following Herren and McMahon [44] to quantify the overall connectivity and stability of microbial associations within each habitat.

Functional potential was predicted with PICRUSt2 [45], with ASVs mapped to KEGG Orthology pathways at level 2. To test the effects of habitat, site, and their interaction on these response variables, we applied linear models followed by Type II Sums-of-Squares Analysis of Variance (ANOVA) using the Anova() function from the car package version 3.1-3 [46]. When significant, *post hoc* pairwise comparisons were conducted using Tukey’s HSD via the emmeans() function (emmeans package version 1.11.2-8 [47]).

To examine community composition and turnover, ASVs were categorized by habitat overlap (unique to one habitat, shared between two, or present in all three). Their proportions were calculated per plot and visualized using stacked bar plots. Taxonomic composition was summarized at the phylum level, with the 20 most abundant phyla visualized by habitat. Phyla with ≥5% mean relative abundance were further analyzed using zero-inflated beta regression models (ZIBMMs) implemented via the glmmTMB package [48]. Type II ANOVA was used to test habitat, site, and interaction effects on phylum-level abundance, followed by *post hoc* comparisons using estimated marginal means. Furthermore, to evaluate community assembly processes, Sloan’s neutral community model (NCM) [49] was applied separately for each habitat. The model relates ASV occurrence frequency to mean relative abundance using nonlinear least squares regression. Model fit (*R*^2^) and estimated migration rates (Nm) were reported. ASVs were classified as fitting within, above, or below neutral expectations, and visualized using log-scaled abundance–frequency plots with fitted curves and 95% confidence intervals.

#### Redefining the *Sphagnum* core bacterial microbiome into host-specific and environmental core groups

Core bacterial microbiomes, in this study, were defined as ASVs with ≥0.1% relative abundance and present in ≥80% of *Sphagnum* samples. This threshold balances ecological relevance and prevalence while minimizing the influence of rare taxa and outlier samples [50–52]. Required ASVs are the total core microbiomes which were categorized as (i) *Sphagnum* unique (SU): present only in *Sphagnum*; (ii) enriched in *Sphagnum* (ES): significantly higher abundance in *Sphagnum* than the surrounding leaf litter and surface organic soil (ZIBMMs with Tukey’s test, *P* < .05); (iii) environmental core (EC): shared across all habitats, not enriched in *Sphagnum*. SU and ES were combined and treated as the host-specific core. Group distributions with each *Sphagnum* core ASV were visualized using heatmaps, and their count and relative abundance proportions were summarized with pie charts. Boxplots were used to compare the difference in the relative abundance of *Sphagnum* core groups between habitats (linear models with Tukey’s test, *P* < .05). The contributions of taxonomic groups to the total core, SU, ES, and EC were assessed based on relative abundance and visualized using bar plots.

To evaluate how *Sphagnum* core microbiomes reflect community structure and habitat divergence, Bray–Curtis dissimilarities were calculated for (i) full microbial communities and (ii) three *Sphagnum* core subsets: total core, environmental core (EC), and host-specific core (SU + ES). Non-metric multidimensional scaling (NMDS) was used to visualize compositional differences across habitats with *k* = 2 dimensions. For each ordination, dispersion within groups (Disp) was quantified using betadisper function of vegan package, and centroid distances (Cdis) between habitat groups were calculated. Variation explained by habitat (*R*^2^) was derived from pairwise PERMANOVA (adonis, 999 permutations). Furthermore, to assess how well core microbiomes represent the full *Sphagnum*-associated community, Mantel tests were performed between Bray–Curtis distance matrices of core subsets and the full community.

#### Determining the ecological and functional contributions of *Sphagnum* core groups to community stability and function

We quantified three indicators to assess how different *Sphagnum* core groups (total core, EC, and *Sphagnum*-specific core (SU + ES)) contribute to all bacterial community stability: (i) average variation degree (AVD), calculated to represent intragroup community convergence; (ii) species association strength, defined as the ratio of positive to negative cohesion values, capturing the balance between cooperative and competitive bacterial interactions; (iii) network robustness, assessed via node removal simulations to estimate the stability of bacterial co-occurrence networks under compositional perturbation. These indicators capture spatial stability across species and environmental gradients within a single sampling period (October). As the study was not temporally replicated, our stability assessment reflects cross-sectional (spatial) rather than temporal consistency of the *Sphagnum* microbiome. The relative abundance of each core group was regressed against its corresponding stability metrics using linear models. Relationships were visualized using scatterplots with 95% confidence intervals. Model fit (*R*^2^) and significance (*F*-test, *P* values) were reported for each association.

Piecewise structural equation modeling (piecewise SEM) [53] was used to determine the different roles of *Sphagnum* core microbiome groups (total core, EC, and *Sphagnum*-specific core) in linking environmental variables (pH and elevation), microbial community convergence in neighboring habitats (litter and soil), overall microbial community structure, and predicted carbon and energy metabolism. Microbial community convergence was quantified by NRI. All community structure of *Sphagnum* microbiomes was represented by the first axis of a NMDS based on Bray–Curtis dissimilarities. Predicted carbon and energy metabolism was required from PICRUSt2 prediction. Fisher’s *C* test (when 0 ≤ Fisher’s *C*/*df* ≤ 2 and 0.05 < *P* ≤ 1.00) was performed to confirm the goodness of the modeling results. The models were gradually revised according to the path significance (*P* < .05) and goodness of the model. The piecewiseSEM and lme4 version 1.1-37 packages were used to conduct the piecewise SEMs [53].

## Results

### Microbial communities of the litter–*Sphagnum*–soil vertical stratification

**Figure 1:**
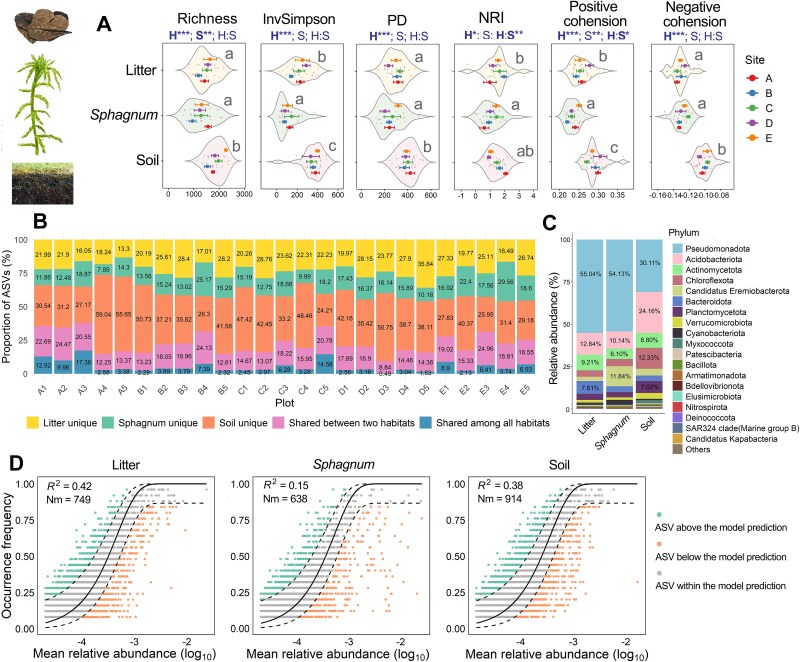
Vertical stratification of microbial communities in the litter–*Sphagnum*–soil system of subtropical peatlands. (A) Bacterial alpha diversity, phylogenetic structure, and co-occurrence network metrics across habitats. Boxplots show richness, inverse Simpson diversity, phylogenetic diversity (PD), net relatedness index (NRI), and cohesion (positive and negative) for each habitat. H: habitat effect; S: site effect. (B) Distribution of ASVs by plot, categorized as unique to each habitat or shared among two or all three compartments. Each plot includes three vertically stratified samples (litter, *Sphagnum*, soil). (C) Relative abundance of the top 20 bacterial phyla across habitats. Proportion values are displayed only for phyla with relative abundance >5%. (D) Neutral community model (NCM) fits for each habitat, showing the relationship between ASV abundance and occurrence frequency. Letters indicate significant differences among habitats (Tukey’s test, *P* < .05); asterisks denote significance of habitat comparisons: ^*^P < .05, ^**^P < .01, ^***^P < .001.

Microbial communities differed significantly across habitats along multiple ecological dimensions within the vertically structured peatland system. Litter and *Sphagnum* hosted comparable levels of richness, PD, and negative cohesion, all of which were lower than in soil. *Sphagnum* supported the lowest microbial evenness (InvSimpson), NRI, and positive cohesion, suggesting a compositionally uneven but phylogenetically overdispersed community shaped by strong deterministic forces. In contrast, soil exhibited the highest values for most diversity and network metrics, except NRI. Functional predictions based on KEGG pathways revealed that soil microbiomes were enriched in genes related to carbon and energy metabolism. Conversely, genes associated with stress adaptation and ecosystem resilience increased progressively from the bottom (soil) to the top (litter) layer (Fig. S2). Beyond vertical stratification, spatial heterogeneity also contributed to community variation, as indicated by significant site effects on richness (Fig. 1A).

Community composition analysis (Fig. 1B) showed that soil harbored the highest proportion of unique ASVs (24.21%–59.04%), followed by litter (13.30%–35.84%) and *Sphagnum* (7.89%–29.56%). Litter and *Sphagnum* contained higher proportions of Pseudomonadota, whereas Acidobacteriota, Chloroflexota, and Planctomycetota were more abundant in soil. Notably, *Ca.* Eremiobacterota were significantly enriched in *Sphagnum* (Fig. 1C). Neutral community model analysis (Fig. 1D) further supported habitat-driven assembly processes: *Sphagnum* communities deviated most from neutral expectations (*R*^2^ = 0.15), indicating strong deterministic selection, while litter (*R*^2^ = 0.42) and soil (*R*^2^ = 0.38) were more consistent with stochastic assembly. Collectively, these findings confirm the strong influence of vertical microenvironmental gradients on the structure and functional potential of peatland microbiomes, and present *Sphagnum* had the distinct hosting microbial communities compared to its surrounding habitats.

### *Sphagnum* core microbiomes and their ecological divergence

**Figure 2:**
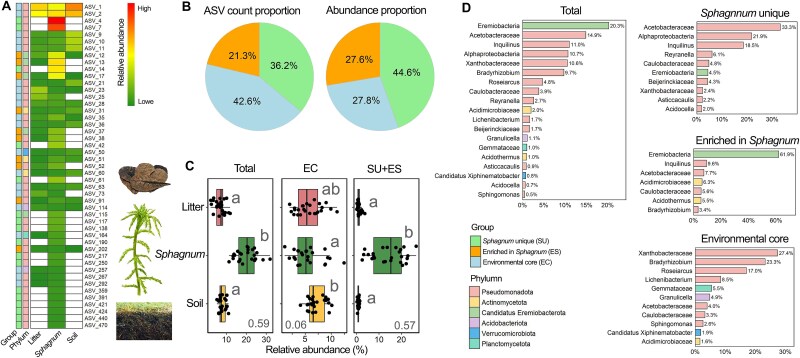
Composition, distribution, and ecological categorization of *Sphagnum* core ASVs in the vertical litter–*Sphagnum*–soil system. (A) Heatmap showing the relative abundance of all *Sphagnum* core ASVs across habitats. ASVs are categorized based on habitat association: *Sphagnum* unique (SU), enriched in *Sphagnum* (ES), and environmental core (EC; shared between soil and litter but not enriched in *Sphagnum*). (B) Proportional contribution of each *Sphagnum* core ASV group based on ASV count (left) and total relative abundance (right). (C) Boxplots of the relative abundance of each ASV group across habitats. *Sphagnum*-specific core microbiomes (SU and ES combined) are compared to environmental core ASVs (EC). Letters indicate significant differences among habitats (Tukey’s test, *P* < .05). Values at the bottom of each panel indicate the effect size (*R*^2^) of habitat from linear models. (D) Taxonomic composition of the total *Sphagnum* core ASVs and each group with the dominant bacterial lineages.

*Sphagnum* core microbiomes were not ecologically homogeneous but instead were stratified into three distinct groups based on their habitat distribution: *Sphagnum* unique (SU), enriched in *Sphagnum* (ES), and environmental core (EC) taxa (Fig. 2A). A heatmap of all 47 identified core ASVs revealed that SU and ES groups were largely restricted to *Sphagnum* tissues, while EC were shared between litter and soil but exhibited low abundance in *Sphagnum* (Table S2). EC comprised 42.6% of the ASV count but contributed only 27.8% of total abundance, whereas SU and ES together represented 57.5% of ASVs and 72.2% of total abundance (Fig. 2B).

Habitat-wise patterns confirmed ecological divergence among these groups (Fig. 2C). The abundance of *Sphagnum*-specific core microbiomes (SU + ES) was strongly shaped by habitat (*R*^2^ = 0.57), whereas EC showed minimal habitat influence (*R*^2^ = 0.06). SU + ES peaked in *Sphagnum* tissues, indicating strong habitat specialization, while EC were significantly more abundant in litter and soil. The overall abundance pattern of core microbiomes was largely driven by SU + ES, potentially masking EC variation.

Taxonomically, *Sphagnum* core microbiomes were dominated by *Ca.* Eremiobacterota and Pseudomonadota (Fig. 2D). SU members were primarily affiliated with Acetobacteraceae (33.3%), Alphaproteobacteria (21.9%), and Inquilinus (18.5%), while ES ASVs were dominated by *Ca*. Eremiobacteria (61.9%), suggesting close host associations. In contrast, EC ASVs were enriched in Xanthobacteraceae (27.4%), Bradyrhizobium (23.3%), and Roseiarcus (17.0%), indicating likely environmental origins. Together, these findings demonstrate clear ecological divergence within the core microbiome, shaped by both environmental context and host-specific filtering.

### Ecological structure and representativeness of *Sphagnum* core microbiomes

**Figure 3:**
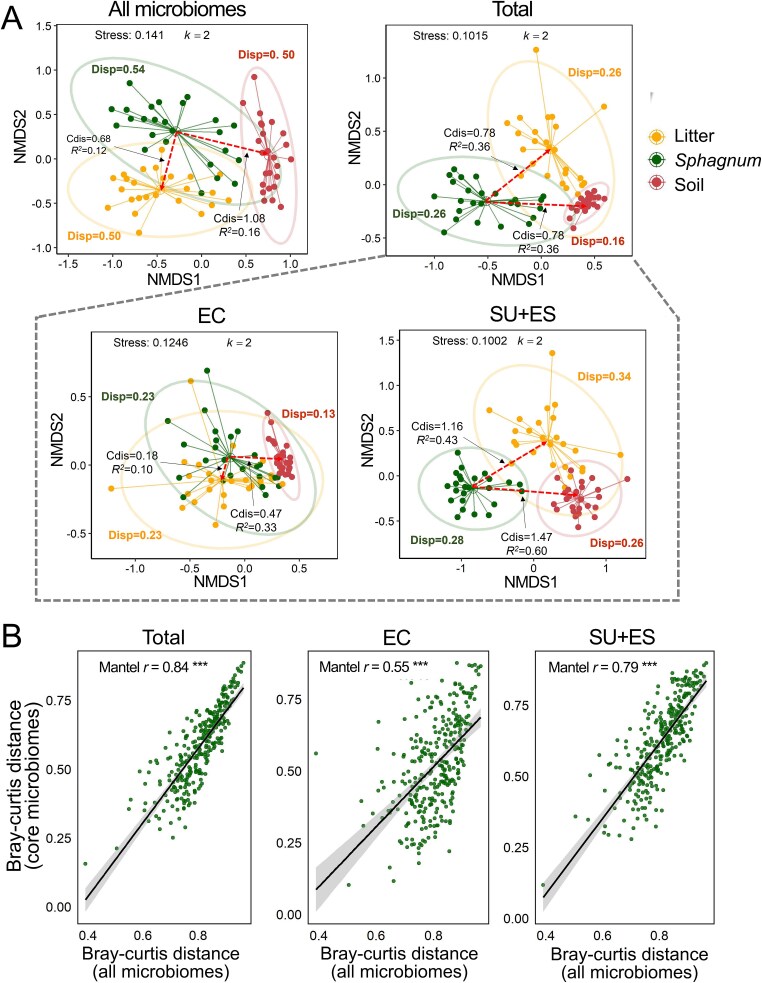
*Sphagnum* core microbiomes reflect both inter-habitat divergence and internal community structure. (A) NMDS ordinations based on Bray–Curtis dissimilarity showing microbial community composition across litter, *Sphagnum*, and soil habitats. Analyses are shown for full microbiomes and three subsets of *Sphagnum* core microbiomes: total core, environmental core (EC), and *Sphagnum*-specific core (SU + ES); Cdis: distance between group centroids; *R*^2^: variation explained by habitat differences from pairwise PERMANOVA; Disp: within-group dispersion. (B) Mantel correlations between Bray–Curtis distances of all and core microbiomes within *Sphagnum* samples, used to evaluate how well each core subset captures the structure of the entire *Sphagnum* microbial community. Asterisks denote significance of habitat comparisons: ^*^P < .05, ^**^P < .01, ^***^P < .001.

To evaluate whether core microbiomes reflect broader community patterns, we compared community structures of full microbiomes and different core groups across habitats. NMDS ordinations revealed clear and consistent habitat separation (Fig. 3A and Table S2). The total *Sphagnum* core microbiome captured the inter-habitat divergence of the full microbiome (PERMANOVA: *R*^2^ = 0.21, *P* = .001; Cdis: *Sphagnum*–litter = 0.68, *R*^2^ = 0.12; *Sphagnum*–soil = 1.08, *R*^2^ = 0.16), but with substantially higher habitat-explained variance (*R*^2^ = 0.51, *P* = .001). *Sphagnum*-specific core (SU + ES) exhibited even stronger habitat differentiation (*R*^2^ = 0.53, *P* = .001; Cdis: *Sphagnum*–litter = 1.16, *R*^2^ = 0.43; *Sphagnum*–soil = 1.47, *R*^2^ = 0.60), indicating strong host specificity. In contrast, EC showed weaker structure differences (*R*^2^ = 0.33, *P* = .001; Cdis: *Sphagnum*–litter = 0.18, *R*^2^ = 0.10; *Sphagnum*–soil = 0.47, *R*^2^ = 0.33) and lower within-group dispersion (*Sphagnum*, Disp = 0.23), and represents generalist or spillover taxa. Furthermore, pH and elevation also contributed to variation in both full and core communities (PERMANOVA: pH, *R*^2^ = 0.035–0.055, *P* < .01; elevation, *R*^2^ = 0.013–0.026, *P* < .05).

Mantel tests further confirmed that core microbiomes captured the internal structure of all *Sphagnum* hosting communities (Fig. 3B). The total core showed a strong correlation with all the microbiomes (Mantel *r* = 0.84, *P* < .001), and SU + ES alone also tracked all community patterns closely (*r* = 0.79, *P* < .001). EC exhibited only moderate correlation (*r* = 0.55, *P* < .001), reinforcing that *Sphagnum*-specific core better represent host-associated microbiome structure and between-habitat divergence. These findings confirm the ecological structuring and representativeness of *Sphagnum* core microbiomes; especially, host-specific core microbiomes best capture both internal community dynamics and cross-habitat divergence.

### Functional contributions of core microbiomes to microbial community stability

**Figure 4:**
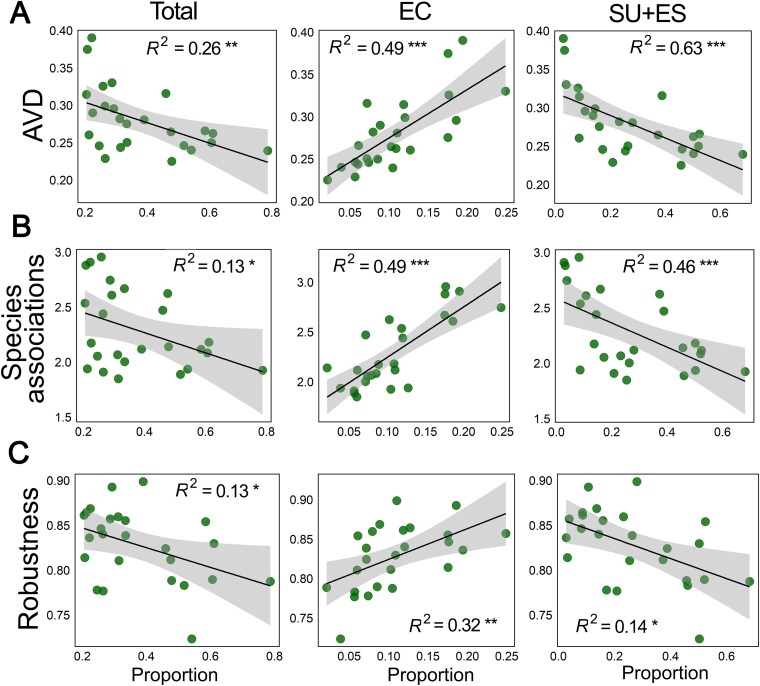
Contributions of *Sphagnum* core microbiomes to microbial community stability. Three indicators of community stability were used to quality the potential contributions of total and each *Sphagnum* core microbiome group: (A) average variation degree (AVD); (B) species association strength, and (C) network robustness. Panels show linear relationships between the relative proportion of core microbiome groups and each stability metric. Shaded areas indicate 95% confidence intervals; *R*^2^ values represent the goodness of fit from linear regression models. Asterisks denote significance of habitat comparisons: ^*^P < .05, ^**^P < .01, ^***^P < .001.

We assessed the contributions of *Sphagnum* core microbiomes to microbial community stability using three ecological indicators: AVD, species association strength, and network robustness (Fig. 4). Core microbiomes showed significant correlations with all stability parameters, but the direction and magnitude varied by core bacterial group. The proportion of *Sphagnum*-specific core taxa (SU + ES) was strongly associated with lower AVD (*R*^2^ = 0.63, *P* < .001), indicating reduced compositional variability and enhanced stability in *Sphagnum* hosting communities. In contrast, EC taxa were positively associated with AVD (*R*^2^ = 0.49, *P* < .001), suggesting that their increase may increase community variability. The total core presents a similar pattern with *Sphagnum*-specific core, a moderate negative relationship with AVD (*R*^2^ = 0.26, *P* < .01; Fig. 4A).

Patterns in species association strength and robustness further revealed divergent effects. Higher proportions of EC taxa were linked to stronger microbial interactions (*R*^2^ = 0.49, *P* < .001; Fig. 4B) and increased robustness (*R*^2^ = 0.32, *P* < .01; Fig. 4C), indicating that EC members support cohesive and resilient community structures. In contrast, SU + ES taxa were associated with lower species association strength (*R*^2^ = 0.46, *P* < .001) and decreased robustness (*R*^2^ = 0.14, *P* < .05; Fig. 4), suggesting looser and more dynamic networks. Together, these results show that *Sphagnum* core microbiomes have contrasting impacts on community stability: EC enhances structural cohesion and resilience but exhibits greater compositional variability, while SU + ES taxa provide stable community composition yet contribute less to network connectivity and robustness. This functional divergence underscores the importance of distinguishing core taxa.

### Differentiated ecological roles of *Sphagnum* core microbiome groups

**Figure 5:**
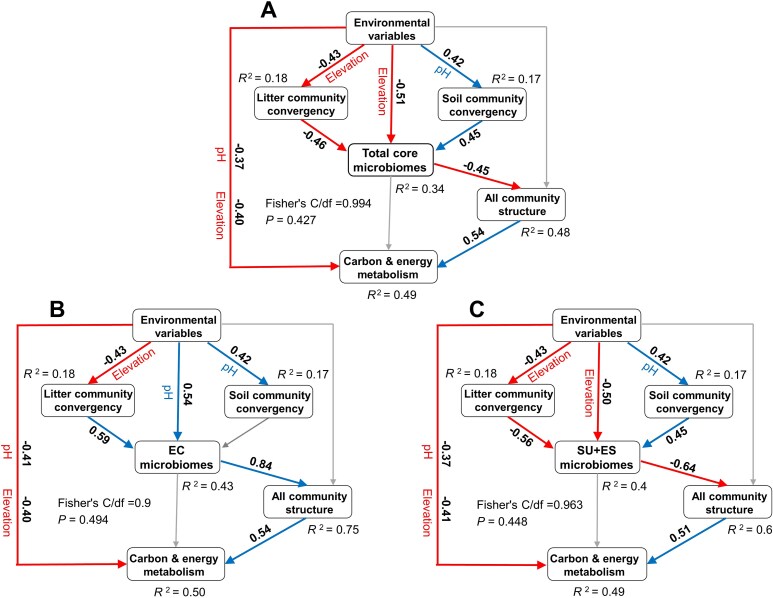
Different ecological roles of *Sphagnum* core microbiome groups. Structural equation models (SEMs) testing the roles of *Sphagnum* core microbiome groups in linking environmental variables (pH and elevation), microbial community convergence in neighboring habitats (litter and soil), overall microbial community structure, and predicted carbon and energy metabolism. Models represent (A) total *Sphagnum* core microbiomes, (B) environmental core microbiomes (EC), and (C) *Sphagnum*-specific core microbiomes (SU + ES). Arrows show standardized path coefficients, where the direction of each relationship is indicated by the sign of the coefficient with positive and negative values. *R*^2^ values denote the proportion of variance explained for each endogenous variable. Fisher’s *C* statistic and associated *P* values indicate overall model fit.

Structural equation models (SEMs) were used to assess how each *Sphagnum* core microbiome links environmental variables, microbial convergence in adjacent habitats, community structure, and predicted metabolic function of all *Sphagnum* microbiomes (Fig. 5). Environmental variables influenced the total *Sphagnum* core microbiome both directly and indirectly by affecting microbial convergence in litter and soil. In turn, total core abundance had a negative effect on overall community structure, which positively influenced the abundance of genes involved in carbon and energy metabolism (0.54, *R*^2^ = 0.49, Fig. 5A). Environmental factors also directly impacted function, with pH (−0.37) and elevation (−0.40) both negatively associated with metabolic potential. Notably, the total core microbiome had no direct effect on function, but influenced it indirectly via restructuring the overall community.

The models for EC and SU + ES also showed similar indirect links to carbon and energy metabolism via overall community structure but revealed distinct ecological pathways (Fig. 5B, C). EC was positively influenced by pH (0.54), whereas elevation was the primary driver of SU + ES abundance (−0.50). Litter community convergence had a strong positive effect on EC (0.59), while soil convergence had no significant impact. In contrast, litter convergence negatively affected SU + ES (−0.56), whereas soil convergence positively influenced it (0.45). EC was strongly and positively associated with overall community structure (0.84), while SU + ES had a negative effect (−0.64). These results indicate that environmental and host-specific core microbiomes have distinct effects on the metabolic potential of *Sphagnum*-associated communities, driven by their divergent roles in structuring overall microbial assemblages.

## Discussion

The vertically structured litter–*Sphagnum*–soil system in subtropical ecosystem harbors distinct microbial communities across layers. Soil supports the highest microbial diversity, likely due to its complex and heterogeneous environment, which includes stable moisture levels, abundant organic matter, and pronounced pH gradients that create a wide range of ecological niches [54–56]. In contrast, both *Sphagnum* and litter exhibit lower microbial diversity. In litter, transient and resource-limited conditions constrain microbial colonization, while in *Sphagnum*, strong host filtering selectively excludes many generalist or weakly adapted taxa [57–59]. Notably, *Sphagnum* microbiomes are structured primarily by deterministic processes such as host selection, in contrast to the more stochastic, dispersal-driven assembly seen in litter and soil. This highlights *Sphagnum*’s role not as a passive substrate, but as an active microbial filter that hosts a uniquely structured and functionally distinct microbiomes.

The higher microbial diversity and stronger network connectivity observed in soil likely reflect its greater environmental heterogeneity and resource complexity compared with *Sphagnum* and litter layers. Soil provides a mosaic of microhabitats with variable oxygen, moisture, and nutrient availability, promoting niche diversification and metabolic interdependence among taxa [60, 61]. In addition, soil receives continuous organic inputs from roots and decomposing litter, sustaining cross-feeding and syntrophic interactions that enhance network complexity [62, 63]. By contrast, the more selective and oligotrophic conditions within *Sphagnum* tissues favor specialized taxa, leading to lower richness and simpler network structures.

**Figure 6:**
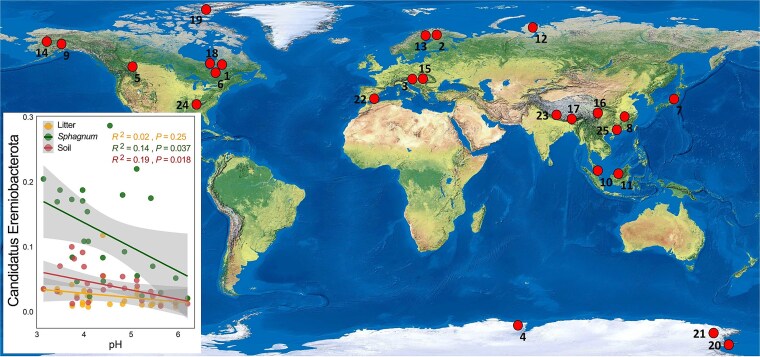
Global occurrence of *Candidatus Eremiobacterota* across extreme ecosystems. Global map showing the presence of *Ca.* Eremiobacterota across 25 published studies encompassing a range of extreme environments, including (1) alpine/high-elevation ecosystem (7,17,23 [94–96]); Arctic/Antarctic (4,19,20,21, [97–100]); bare environments (5, [101]); boreal forest (1,6,9,18, [34, 102–104]); cave (22 [105]); mining areas (8,24,25, [106–108]); peatland (3,10,11,13,15,16, [109–114]); tundra (2,12,14, [65, 115, 116]). The inset graph shows the relationship between pH and the relative abundance of *Ca.* Eremiobacterota in litter, *Sphagnum*, and soil from the studied subtropical peatland system. A significant negative association was found in *Sphagnum* (*R*^2^ = 0.14, *P* = .037) and soil (*R*^2^ = 0.19, *P* = .018), but not in litter (*R*^2^ = 0.02, *P* = .25), suggesting that lower pH environments—especially within *Sphagnum* tissues—favor the enrichment of this group.

A striking finding of our study is the strong enrichment of *Ca.* Eremiobacterota in *Sphagnum* tissues, where it constitutes >20% of the total core microbiome. These taxa dominate the enriched core of *Sphagnum*, accounting for >60% of host-selected ASVs, yet remain rare in adjacent soil and litter. Such a distribution pattern suggests that *Sphagnum* acts not merely as a passive substrate but as an active microbial filter, selectively recruiting taxa like *Ca.* Eremiobacterota that are ecophysiologically adapted to its acidic, oligotrophic, and water-saturated niche. Members of this phylum are known to play critical roles in carbon cycling, nitrogen fixation, and energy flow, particularly in acidic and oligotrophic environments [64, 65]. Genomic evidence suggests that some lineages harbor genes for high-affinity hydrogen oxidation, anoxygenic photosynthesis, and nitrogen fixation, enabling them to persist in nutrient-limited conditions and potentially supplement the host’s carbon and nitrogen demands [64, 66, 67]. Their ability to oxidize atmospheric H₂ and fix CO₂ through autotrophic pathways may enhance microbial-driven carbon input in the *Sphagnum* phyllosphere, while diazotrophic capabilities may partially compensate for low inorganic nitrogen availability in subtropical peatlands. Furthermore, *Ca.* Eremiobacterota has also been reported in alpine, Arctic/Antarctic, bare, boreal, cave, mining, and tundra ecosystems (Fig. 6), highlighting their ecological versatility and contribution to biogeochemical fluxes in diverse, climate-sensitive ecosystems. As global change accelerates pH decline and environmental stress in these habitats [68, 69], understanding how *Sphagnum* recruits or maintains these beneficial taxa will be essential for predicting the resilience of peatland microbiomes. More studies on *Ca.* Eremiobacterota are crucial for understanding microbial contributions to ecosystem resilience and biogeochemical feedbacks under global change.

The extent to which core microbiomes reflect broader community patterns is a critical question in microbial ecology, particularly for ecosystems facing rapid environmental change [27, 28, 70]. The total *Sphagnum* core microbiomes captured habitat-level divergence more effectively than the full microbiomes (*R*^2^ = 0.51 vs. 0.21), confirming its value as a representative subset of overall host performance [71–73]. This elevated explanatory power supports that core microbiomes, particularly those shaped by *Sphagnum* filtering, are reliable indicators of deterministic assembly processes in peatlands, where *Sphagnum* are fundamental ecosystem engineers creating the low-pH, anaerobic, and oligotrophic conditions essential for peatland carbon storage and ecosystem resilience [74–77]. Different from host-specific core group, EC showed weak habitat structuring, low dispersion, and only moderate correlation with full community patterns—consistent with their role as generalist or spillover taxa likely introduced via environmental exposure [78]. These results support our view that not all core taxa are ecologically equivalent, and that host-enriched core groups better reflect the deterministic processes shaping microbiome assembly in *Sphagnum*.

*Sphagnum* core microbiomes have contrasting effects on community stability: host-specific core taxa reduce compositional variability, stabilizing community structure, but weaken microbial connectivity and robustness, while EC taxa increase variability yet enhance interaction strength and network resilience—highlighting their complementary roles in shaping peatland microbiome dynamics. This divergence may reflect distinct ecological roles and assembly histories. Host-filtered taxa likely experience selective pressures for functional specialization and compatibility, leading to dominance but reduced network interdependence. Conversely, EC taxa, being generalist and environmentally dispersed, may engage in broader metabolic interactions and occupy redundant niches, promoting network-level resilience [79, 80]. These patterns align with current ecological theories that specialist-dominated systems often exhibit compositional stability but reduced robustness under perturbation, whereas generalist-rich systems provide greater flexibility and structural resilience [81–83]. Together, these results highlight the importance of distinguishing host-specific and environmental core microbiomes, as they represent functionally divergent strategies that differentially contribute to community stability and resilience.

Both EC and *Sphagnum*-specific core groups influenced carbon and energy metabolism indirectly through their contrasting effects on overall community structure. Among these, *Ca.* Eremiobacterota stands out as a dominant and functionally significant core lineage, likely contributing directly to carbon and nitrogen cycling while also shaping the compositional balance of the *Sphagnum* microbiome. Thus, microbiome function likely arises from an interplay between the specific activities of key core taxa and the emergent properties of community organization [84–86]. While PICRUSt2 offers useful insights into potential functions, its 16S-based predictions do not directly capture gene content or activity [45]. Future metagenomic and metatranscriptomic analyses could help disentangle the relative contributions of core taxa and community-level interactions to microbiome function. Core microbiome groups were shaped by distinct environmental drivers. EC abundance increased with pH, consistent with its origin from neighboring habitats (i.e. litter and soil), where pH strongly governs microbial distribution [87, 88]. In contrast, *Sphagnum*-specific core abundance showed no relationship with pH but declined with elevation. This pattern may reflect elevation-driven shifts in host–microbe interactions along vertical and climatic gradients, a hypothesis that warrants direct testing through future measurements of host functional traits [89].

Furthermore, core microbiome abundance was shaped by convergence in surrounding communities, reflecting distinct ecological origins. EC abundance was positively associated with litter convergence, indicating that stronger environmental filtering in litter promotes a more compatible and predictable source pool for environmentally derived taxa [90]. EC abundance was unaffected by soil convergence, suggesting limited recruitment from soil due to stronger niche differences or dispersal constraints [91, 92]. In contrast, *Sphagnum*-specific core abundance was negatively related to litter convergence, possibly due to competitive exclusion or biotic filtering from phylogenetically clustered litter communities. Its positive association with soil convergence suggests that more convergent soil communities provide stable and compatible microbial pools for recruitment into the *Sphagnum* host [93]. These patterns highlight differing source dynamics and assembly processes between environmental and host-specific core microbiomes. To confirm the temporal stability and ecological roles of these core taxa, future studies should incorporate longitudinal sampling across months, seasons, and years, enabling a deeper understanding of their persistence and functional significance over time.

## Conclusion

This study reveals that *Sphagnum* in subtropical peatlands hosts compositionally and functionally distinct microbiomes shaped by deterministic filtering. By redefining the core microbiome into host-specific and environment-derived components, we provide evidence for potential contrasting roles: host-specific cores may help stabilize community composition, while environmental cores enhanced network resilience. These core groups appeared to respond differently to environmental gradients, indicating that global change could shift their balance and impact peatland microbial stability and function. Overall, our work advances understanding of host–microbe interactions in climate-sensitive ecosystems and underscores that recognizing functional differences among core members is essential for predicting microbiome assembly and ecosystem responses to environmental change.

## Supplementary Material

Supporting_Matierals_ISME_ycaf221

## Data Availability

Amplicon sequencing data from this study are available at the NCBI Sequence Read Archive (SRA) under the BioProject PRJNA1283477.
