# Supplementary material for: Host-specific and environmental core bacteria differentially shape the stability and function of the Sphagnum phyllosphere
Source: ISME Commun. 2025 Nov 25;5(1):ycaf221. doi: 10.1093/ismeco/ycaf221 (PMC12713642; doi:10.1093/ismeco/ycaf221)
Supplement: Supporting_Matierals_ISME_ycaf221 [file supporting_matierals_isme_ycaf221.docx]

**Supporting materials**

**Table S1**. Effects of habitat and site on bacterial phyla abundance (≥5%) in a peatland vertical system

|  | **Pseudomonadota** | | **Acidobacteriota** | | **Actinomycetota** | | **Candidatus Eremiobacterota** | | **Chloroflexota** | | **Bacteroidota** | | **Planctomycetota** | |
| --- | --- | --- | --- | --- | --- | --- | --- | --- | --- | --- | --- | --- | --- | --- |
| **Effects of habitat and site on phylum abundance (Type II ANOVA)** | | | | | | | | | | | | | | |
|  | Chisq | *P* | Chisq | *P* | Chisq | *P* | Chisq | *P* | Chisq | *P* | Chisq | *P* | Chisq | *P* |
| **Habitat** | 127.89 | **<0.001** | 122.02 | **<0.001** | 17.51 | **<0.001** | 96.50 | **<0.001** | 48.22 | **<0.001** | 44.75 | **<0.001** | 51.09 | **<0.001** |
| **Site** | 2.49 | **<0.001** | 3.54 | 0.471 | 4.28 | 0.369 | 42.56 | **<0.001** | 13.78 | **0.008** | 23.68 | **<0.001** | 5.34 | 0.25 |
| **Habitat:Site** | 16.44 | **0.036** | 1.74 | 0.988 | 25.17 | **0.001** | 11.17 | 0.192 | 2.55 | 0.960 | 5.10 | 0.747 | 10.81 | 0.21 |
| **Post Hoc Tukey comparison of Habitat effects (estimated marginal means & grouping)** | | | | | | | | | | | | |  |  |
|  | emmean | Group letter | emmean | Group letter | emmean | Group letter | emmean | Group letter | emmean | Group letter | emmean | Group letter | emmean | Group letter |
| **Litter** | 0.198 | b | -1.91 | b | -2.35 | b | -2.35 | a | -3.26 | a | -2.57 | b | -3.41 | a |
| **Sphagnum** | 0.164 | b | -2.22 | a | -2.72 | a | -2.72 | b | -3.06 | a | -3.38 | a | -3.41 | a |
| **Soil** | -0.835 | a | -1.14 | c | -2.36 | b | -2.36 | a | -2.04 | b | -3.38 | a | -2.58 | b |

**Table S2**. PERMANOVA results for drivers of microbial community variation across all microbiomes and each group of *Sphagnum* core microbiomes

|  | **Df** | **SumOfSqs** | ***R*^2^** | **F** | **Pr(>F)** |
| --- | --- | --- | --- | --- | --- |
| **all** |  |  |  |  |  |
| Elevation | 1 | 0.67 | 0.026 | 2.962 | 0.004 ** |
| pH | 1 | 1.18 | 0.047 | 5.235 | 0.001 *** |
| Habitat | 2 | 5.38 | **0.214** | 11.957 | 0.001 *** |
| Site | 4 | 2.28 | 0.090 | 2.530 | 0.001 *** |
| Habitat:Site | 8 | 2.64 | 0.105 | 1.463 | 0.004 ** |
| **Total core** |  |  |  |  |  |
| Elevation | 1 | 0.13 | 0.015 | 3.078 | 0.031 * |
| pH | 1 | 0.37 | 0.043 | 8.709 | 0.001 *** |
| Habitat | 2 | 4.44 | **0.509** | 51.749 | 0.001 *** |
| Site | 4 | 0.53 | 0.061 | 3.097 | 0.001 *** |
| Habitat:Site | 8 | 0.75 | 0.086 | 2.195 | 0.002 ** |
| **EC** |  |  |  |  |  |
| Elevation | 1 | 0.08 | 0.015 | 2.136 | 0.049 * |
| pH | 1 | 0.29 | 0.055 | 7.863 | 0.001 *** |
| Habitat | 2 | 1.72 | **0.330** | 23.479 | 0.001 *** |
| Site | 4 | 0.47 | 0.090 | 3.196 | 0.001 *** |
| Habitat:Site | 8 | 0.53 | 0.101 | 1.796 | 0.017 * |
| **SU+ES** |  |  |  |  |  |
| Elevation | 1 | 0.20 | 0.013 | 2.737 | 0.038 * |
| pH | 1 | 0.52 | 0.035 | 7.089 | 0.001 *** |
| Habitat | 2 | 7.88 | **0.528** | 53.648 | 0.001 *** |
| Site | 4 | 0.80 | 0.054 | 2.729 | 0.002 ** |
| Habitat:Site | 8 | 1.25 | 0.084 | 2.130 | 0.002 ** |

Notes: Permutational multivariate analysis of variance (PERMANOVA) based on Bray–Curtis dissimilarity quantifying the effects of elevation, pH, habitat type (Part/Habitat), site, and their interactions on microbial community composition. Results are shown for all microbiomes, total Sphagnum core ASVs, environmental core (EC) ASVs, and Sphagnum-specific core ASVs (SU + ES). The proportion of variance explained (R²), F-statistics, and associated P-values are presented. Significant terms are indicated: : *P < 0.05, ** P < 0.01, ***P < 0.001.


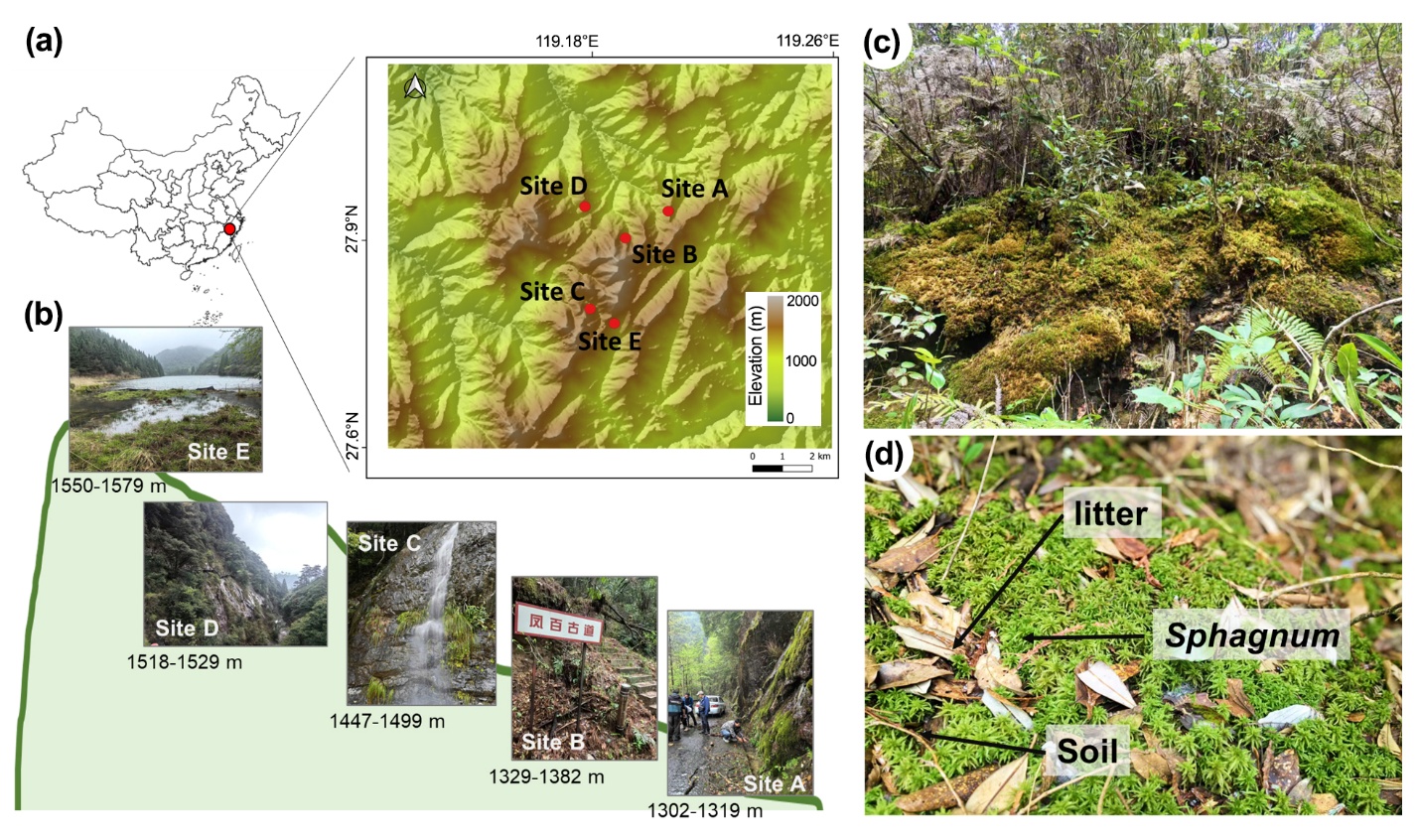


**Figure S1** Study site overview and sampling design in the Baishanzu National Nature Reserve. (a) Location of Baishanzu National Nature Reserve in southeastern China, with the five sampling sites (A–E); (b) Photographs of each site arranged by elevation (1302–1579 m), illustrating representative habitat conditions; (c) Typical *Sphagnum*-dominated peatland observed at the study sites; (d) The three microhabitats sampled at each site: overlying litter, living *Sphagnum* moss, and underlying soil. This design enables investigation of microbial community stratification across vertical layers in *Sphagnum* peatlands.


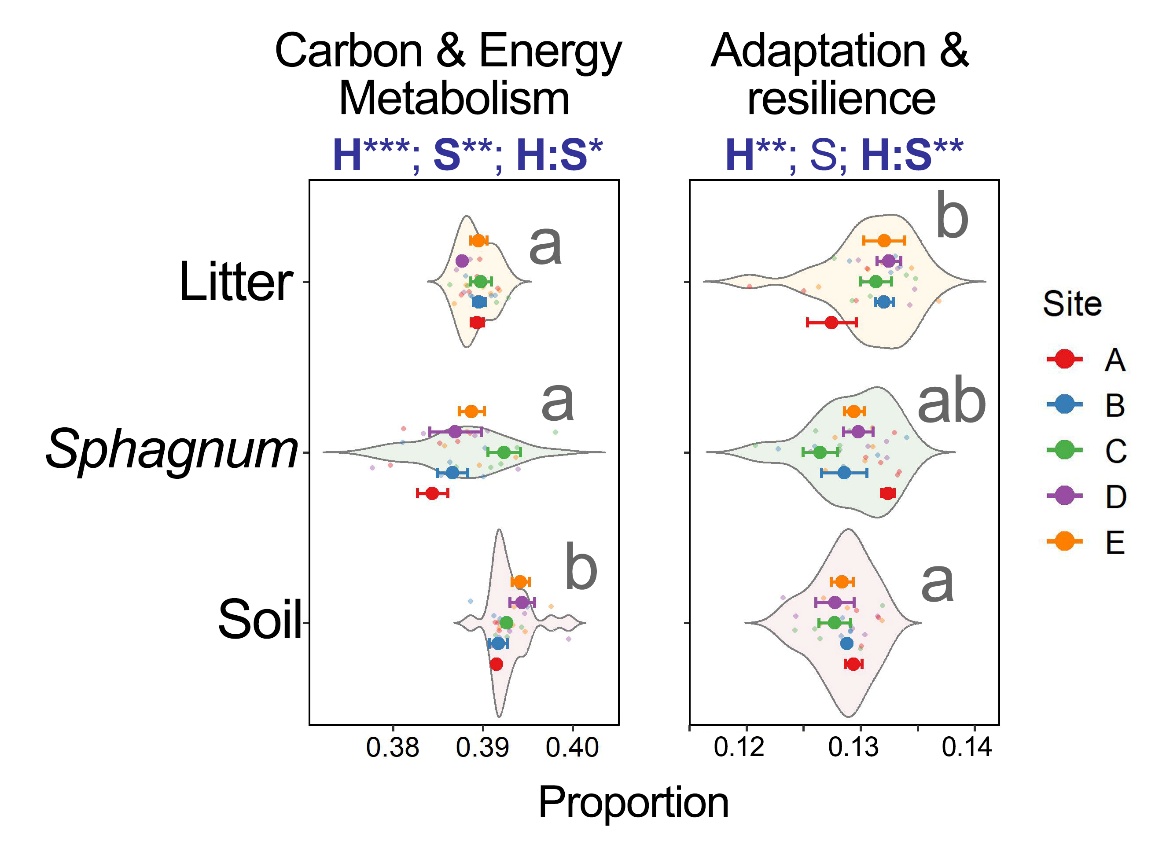


**Figure. S2** Differential functional potentials of microbiomes across vertical peatland habitats. Predicted proportions of microbial genes related to (left) carbon and energy metabolism and (right) stress adaptation and ecosystem resilience across litter, *Sphagnum*, and soil microbiomes. Functional profiles were inferred using PICRUSt2 and mapped to KEGG Orthology level 2 (L2) pathways. Carbon & Energy Metabolism includes: carbohydrate metabolism, lipid metabolism, energy metabolism, amino acid metabolism, and nucleotide metabolism. Stress Adaptation & Resilience includes: xenobiotics biodegradation and metabolism, environmental adaptation, signal transduction, and membrane transport. Violin plots show the distribution of site-level values (dots and colored error bars) across habitats. Letters indicate significant pairwise differences between habitats based on Tukey’s HSD (P < 0.05). Effects of habitat (H), site (S), and their interaction (H:S) were tested using Type II ANOVA. Significance is denoted as: *P < 0.05, **P < 0.01, ***P < 0.001.
